# Subarachnoid Hemorrhage Associated with Ventricular Fibrillation and Out-of-Hospital Cardiac Arrest

**DOI:** 10.1155/2009/375676

**Published:** 2009-07-20

**Authors:** Hidetada Fukushima, Kenji Nishio, Kazuo Okuchi

**Affiliations:** Department of Emergency and Critical Care Medicine, Nara Medical University, Nara 634-8522, Japan

## Abstract

Aneurysmal subarachonoid hemorrhage (SAH) is a common cause of out-of-hospital cardiac arrest (OHCA). Even after successful resuscitation, most of these SAH patients suffer brain death or enter a vegetative state. To our knowledge, survival without neurological damage from SAH following OHCA is quite a rare event. We treated a case of SAH who presented with OHCA and survived without neurological sequelae. A 50-year-old woman presented with ventricular fibrillation (VF), and was successfully resuscitated before hospital arrival. Since there was no evidence of acute coronary syndrome, a head CT scan was performed and established the diagnosis of SAH. On arrival, she was comatose, however, 3 hours after admission, her neurological status recovered. She underwent treatment for the ruptured aneurysms and was discharged from hospital without any neurological deficits.

## 1. Introduction

Aneurysmal subarachnoid hemorrhage (SAH) is a well-known cause of sudden cardiac arrest. It was reported that 4% of patients with SAH experience out-of-hospital cardiac arrest (OHCA) [1]. These OHCAs induced by SAH may present with ventricular arrhythmia and be misdiagnosed as acute coronary OHCA [2]. Identifying the cause of cardiac arrest is essential for guiding the appropriate managements. In this report, we present a case of SAH who survived an episode of OHCA without neurological sequelae, and we discuss the management of this group of OHCA patients.

## 2. Case Presentation

**Figure 1:**
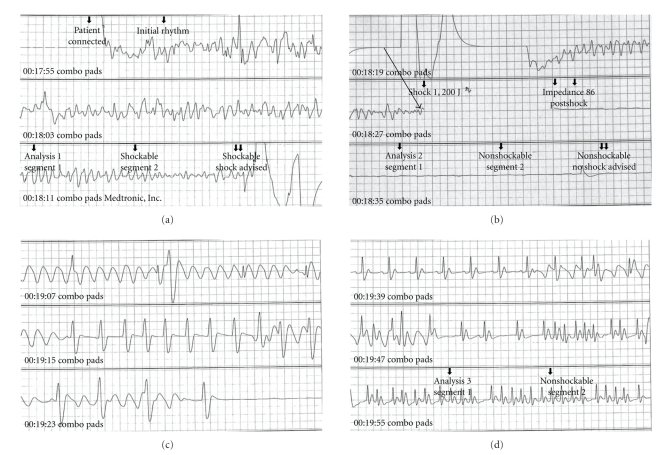
Electrocardiography of the presented case recorded by the automated external defibrillator (AED). (a) The initial heart rhythm was ventricular fibrillation (VF). (b)Successful defibrillation using 200 joules (arrow). VF was converted to asystole. (c) Spontaneous heart rhythm was recognized among the waves by chest compressions. (d) The patient's heart started beating irregularly.

**Figure 2:**
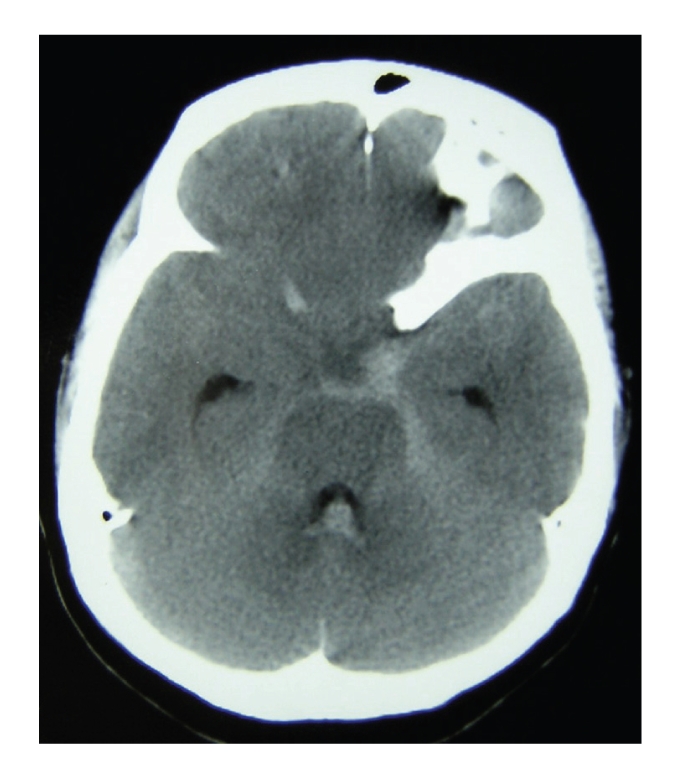
A head CT scan of the presented case. A head CT scan showed subarachnoid hemorrhage in the supracella cistern with hematoma in the fourth ventricle. Cerebral edema with sulcal effacement is also recognized.

A 50-year-old woman with no prior medical history had complained of severe headaches for three weeks before she collapsed at home. The local emergency medical service (EMS) found that she was in cardiac arrest. Cardiopulmonary resuscitation was started, and an automated external defibrillator (AED) was used to shock the patient. Soon after the first shock, she regained spontaneous circulation but remained unconscious. Later analysis of the AED record revealed that her initial heart rhythm was ventricular fibrillation (Figure 1). Call-arrival time of EMS was 8 minutes, and the estimated time of cardiac arrest was about 5 minutes. Neither endotracheal intubation nor intravenous access was performed by EMS personnel. On arrival at the emergency department, she was flaccid, comatose (Glasgow Coma Scale of 3), with deep spontaneous breathing, but hemodynamically stable without administration of vasopressors. Chest X-ray, electrocardiography, echocardiography, and laboratory tests were unremarkable. Since there was no evidence of cardiogenic cardiac arrest, a head CT scan was performed and revealed diffuse high density in the subarachnoid space (Figure 2). SAH was considered the cause of OHCA. According to the consulting neurosurgeon, immediate surgical intervention was not indicated because she was in postresuscitation period and remained comatose. She was admitted to intensive care unit for hemodynamic monitoring with mechanical ventilation. However, 3 hours after admission to the intensive care unit, her neurologic condition improved spontaneously to a Glasgow Coma Scale of 9 (E4V1M4). A cerebral angiography was arranged and revealed aneurysms at the bifurcation of the left internal carotid artery and posterior communicating (IC-PC) artery and the left anterior choroidal artery. She underwent a craniotomy for clipping of the raptured aneurysms immediately. A day after the surgery, she recovered sufficiently to obey verbal commands. She had no neurological deficits at discharge from the hospital.

## 3. Discussion

The prognosis of SAH resuscitated from OHCA is extremely poor; most of these results in brain death, vegetative state, or severe brain damage with lower survival rate. Survival from SAH following OHCA without neurological deficit is quite rare. The mechanism whereby SAH induces cardiac arrest is attributed to sudden increase of intracranial pressure caused by massive SAH, resulting in brain stem compression, brain herniation, and subsequent respiratory and cardiac arrest. Another mechanism is excessive catecholamines release, which causes heart failure or fatal ventricular arrhythmia [3]. Among all OHCA victims with ventricular arrhythmia, acute coronary syndrome is the most likely cause. The frequency of SAH-associated OHCA that occurs with ventricular arrhythmia is less than 1% [1]. These OHCA victims with SAH should be managed differently from acute coronary victims, especially consideration should be given to neurosurgical intervention rather than conventional hemodynamic monitoring or therapeutic hypothermia. The sudden onset of severe headache is a typical symptom of SAH and the key for the diagnosis. However, it was reported that about one-half of the SAH-associated OHCA victims suddenly lose consciousness without any prodromal symptom [1]. The diagnosis is not difficult if CT scan is available. The usefulness of head CT scans for resuscitated OHCA patients remains controversial [4], and delaying therapeutic hypothermia for head CT in all patients is unwarranted; however, it is critical to rule out intracranial hemorrhage since there is no evidence for benefit of therapeutic hypothermia for this group of OHCA patients [5]. Additionally, therapeutic hypothermia increases bleeding tendency, which is detrimental to patients with intracranial bleeding [6]. Consideration for neurosurgical intervention should be the top priority.

In conclusion, SAH-induced ventricular arrhythmia could result in sudden cardiac arrest in out of the hospital setting. Prompt resuscitation and neurosurgical intervention is essential for good neurologic outcome, especially for patients with improving neurological status after restoring spontaneous circulation. The diagnosis should not be delayed.
